# Transcriptomic Insights Into the Evolution of Snake Venom: Mechanisms, Diversity, and Adaptation

**DOI:** 10.1155/sci5/8813839

**Published:** 2026-05-21

**Authors:** Fajar Sofyantoro, Wisnu Ananta Kusuma, Setyanto Tri Wahyudi, Favorisen Rosyking Lumbanraja, Hendra Rahmawan, Heru Cahya Rustamaji, Dhadhang Wahyu Kurniawan, Undri Rastuti, Wahyu Aristyaning Putri, Wiko Arif Wibowo, Dwi Sendi Priyono, Donan Satria Yudha, Slamet Raharjo, Tri Rini Nuringtyas

**Affiliations:** ^1^ Department of Tropical Biology, Faculty of Biology, Universitas Gadjah Mada, Yogyakarta, Indonesia, ugm.ac.id; ^2^ Department of Computer Science, School of Data Science, Mathematics, and Informatics, IPB University, Bogor, Indonesia, ipb.ac.id; ^3^ Tropical Biopharmaca Research Center, IPB University, Bogor, Indonesia, ipb.ac.id; ^4^ Bioinformatics Study Program, Faculty of Mathematics and Natural Sciences, IPB University, Bogor, Indonesia, ipb.ac.id; ^5^ Department of Physics, Faculty of Mathematics and Natural Sciences, IPB University, Bogor, Indonesia, ipb.ac.id; ^6^ Department of Computer Science, Faculty of Mathematics and Natural Sciences, Universitas Lampung, Lampung, Indonesia, unila.ac.id; ^7^ Department of Informatics, Faculty of Industrial Technology, UPN Veteran Yogyakarta, Yogyakarta, Indonesia; ^8^ Department of Pharmacy, Faculty of Health Sciences, Universitas Jenderal Soedirman, Purwokerto, Central Java, Indonesia, unsoed.ac.id; ^9^ Department of Chemistry, Faculty of Mathematics and Natural Sciences, Universitas Jenderal Soedirman, Purwokerto, Central Java, Indonesia, unsoed.ac.id; ^10^ Department of Internal Medicine, Faculty of Veterinary Medicine, Universitas Gadjah Mada, Yogyakarta, Indonesia, ugm.ac.id; ^11^ Research Center for Biotechnology, Universitas Gadjah Mada, Yogyakarta, Indonesia, ugm.ac.id

**Keywords:** adaptation, evolution, multiomics, snake venom, transcriptomics

## Abstract

Snake venoms are evolutionarily refined biochemical arsenals composed of diverse toxins with complex functional roles in predation, defense, and competition. Over the past 2 decades, transcriptomic approaches have transformed venom research by enabling high‐resolution insights into gene expression dynamics, molecular diversity, and the evolutionary mechanisms driving venom variation across lineages. In this review, we present a comprehensive synthesis of snake venom transcriptomics literature and propose a conceptual framework structured around three major axes: (1) gene family expansion through duplication and neofunctionalization; (2) regulatory complexity encompassing transcriptional, posttranscriptional, and epigenetic modulation; and (3) ecological selection pressures shaping venom profiles in response to diet, habitat, and interspecific interactions. We integrate findings from diverse taxa and technologies, including bulk RNA sequencing, long‐read transcriptomics, and spatial or single‐cell approaches, to highlight progress and gaps in current knowledge. A bibliometric analysis of 358 studies from 2002 to 2024 reveals significant growth in the field, with key contributions from institutions in the United States, Brazil, and Australia. Despite this progress, transcriptomic research remains geographically and taxonomically biased, with challenges in toxin annotation, data standardization, and integrative multiomics still unresolved. We conclude by emphasizing the growing role of integration with other omics approaches, advancements in single‐cell transcriptomics, and the emerging potential of computational modeling in reconstructing venom evolution.

## 1. Introduction

Snake venom fulfils an essential adaptive function within predator–prey dynamics, acting as a biochemical tool that enables snakes to effectively incapacitate and capture their prey [1, 2]. The composition and strength of venom are often finely tuned to align with the specific prey encountered by various snake species [3]. For example, front‐fanged snakes such as vipers have evolved venoms containing compounds like metalloproteinases and cobratoxin, which target a wide array of both vertebrate and invertebrate prey [4]. This adaptability is also reflected in the coevolutionary interactions between venomous snakes and their prey, where prey species develop resistance mechanisms, prompting an evolutionary arms race. A case in point is the interaction between rattlesnakes and mammalian prey such as ground squirrels, which demonstrates localized adaptations in venom potency and resistance, underscoring the complexity of coevolution [5–7]. Furthermore, the molecular mechanisms that drive venom evolution are diverse, with gene duplication and positive selection fostering the neofunctionalization of toxin genes, as observed in the king cobra (*Ophiophagus hannah*) [8]. This evolutionary process is further exemplified by the prey‐specific toxicities found in rear‐fanged snakes, where certain toxins exhibit heightened effectiveness against particular prey types, such as lizards or mammals [3]. Moreover, the importance of snake venom adaptation extends beyond predation; in some instances, it has evolved to act as a defensive tool. Spitting cobras, for instance, produce venom components that cause pain to deter predators [9]. The adaptive role of snake venom in predator–prey interactions illustrates the complex evolutionary forces shaping these biological systems, driving the diversification and specialization of venom components to satisfy the ecological requirements of different snake species [10].

Transcriptomics has become an indispensable tool for investigating gene expression in snake venom glands, offering essential insights into the evolutionary, ecological, functional, and pharmacological aspects of animal venom. Through transcriptome sequencing of venom glands, researchers can thoroughly analyze and catalog toxin composition across various snake species, including those producing limited venom volumes, such as small rear‐fanged snakes [11]. This technique has uncovered notable variability in gene expression and coding sequences that account for functional differences in venom across ecological and evolutionary contexts [12]. For example, de novo assembly and examination of the venom gland transcriptome of *Calloselasma rhodostoma* identified 92 unique transcripts across 16 toxin families, illustrating the remarkable complexity and diversity of venom components [13, 14]. Comparative transcriptomic analyses, such as those performed on *Naja kaouthia* from different regions, further highlighted the extensive diversity of toxin genes and their geographically specific variations, which is vital for improving antivenom formulations [15]. These technological advances also emphasize the critical role of transcriptomics in understanding the dynamics of venom replenishment and rapid synthesis of venom proteins postdepletion [16]. Thus, transcriptomics offers a robust framework to explore the genetic foundations of venom diversity, with implications for ecological studies and therapeutic applications. Importantly, connecting molecular venom profiles to clinical outcomes is increasingly relevant because snakebite envenomation frequently produces severe systemic complications, including renal toxicity/acute kidney injury, that strongly influence morbidity and mortality in affected regions [17].

This review aims to synthesize current knowledge on the evolutionary insights derived from transcriptomic analyses of snake venoms. It specifically explores how transcriptomic data elucidate the processes of toxin gene family expansion, regulatory adaptation, and interspecies diversity in venom composition. To provide a more comprehensive and structured understanding of venom evolution, we introduce a unified conceptual model that organizes these findings along three thematic axes: (1) gene family expansion, (2) regulatory complexity, and (3) ecological selection pressures. This framework contextualizes past discoveries, highlights the limitations and challenges of current transcriptomic approaches, and offers a lens through which future research in snake venomics can be systematically guided and interpreted.

## 2. Bibliometrics Landscape of Snake Venom Transcriptomics

To contextualize the need for a structured conceptual framework, we conducted a bibliometric analysis of the global research landscape on snake venom and transcriptomics. The keywords “(transcriptome OR transcriptomics) AND (snake AND venom)” were applied within the fields of “Article Title, Abstract, Keywords” in the Scopus database. Scopus was chosen for its comprehensive coverage of high‐quality, peer‐reviewed literature and its exclusion of predatory journals, thus ensuring the reliability and credibility of the retrieved data. The search, conducted on December 6, 2024, yielded a total of 358 documents published between 2002 and 2024 (Supporting Table S1). The analysis revealed that the majority of documents were research articles (*n* = 295; 82.4%), followed by reviews (*n* = 39; 10.9%), editorials (*n* = 12; 3.4%), conference papers (*n* = 4; 1.1%), and others (*n* = 8; 2.2%). The modest volume of transcriptomics research on snake venom, compared with other fields, reflects several persistent challenges. First, the biochemical complexity and high interspecific variability of venom components, even within a single species, complicate transcriptomic analyses [18]. Second, regulatory barriers to wildlife collection and venom transfer, especially in biodiversity‐rich but resource‐limited countries, restrict sample access and international collaboration [19]. Third, the annotation of toxin genes remains labor‐intensive, with many bioinformatic pipelines overestimating gene diversity due to misclassification of short transcripts [20]. In addition, tools for venom gland transcriptome assembly and analysis are less standardized and accessible than those used in other model systems [12]. Moreover, limited integration of transcriptomic and proteomic data, which is crucial for accurate toxin profiling, further constrains insights into venom biology [18]. Research in this area also faces funding limitations and lacks infrastructure in many endemic regions, especially for neglected species [19]. Finally, although multiomics platforms are emerging, their adoption across snake venom studies remains uneven and institutionally concentrated [21].

### 2.1. Publication Trend Over Time

**FIGURE 1:**
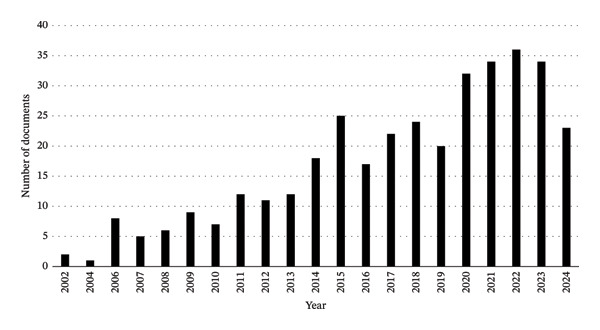
Annual trends in the number of published documents related to snake venom and transcriptomics from 2002 to 2024.

Figure 1 illustrates a clear upward trend in research output from 2002 to 2024. In the early period (2002–2010), fewer than 10 papers were published annually, reflecting early barriers in applying transcriptomics to venom studies and limited access to next‐generation sequencing (NGS). From 2011 onward, a steady rise is observed, coinciding with the adoption of NGS and more robust bioinformatics tools. The number of publications peaked in 2022, with 36 articles, highlighting increased interest and capacity in this field.

### 2.2. Geographic and Institutional Concentration of Transcriptomics Output

As shown in Tables 1 and 2, countries with the highest publication output also host the most productive institutions. The United States leads with 116 publications (32.40%), supported by institutions such as Florida State University and Clemson University. Brazil ranks second with 78 publications (21.8%), driven by the research output of Instituto Butantan (*n* = 49; 13.69%) and the Universidade de São Paulo (*n* = 26; 7.26%). Spain and Australia also contribute substantially, with 33 and 50 publications, respectively. Their productivity is largely attributed to the Consejo Superior de Investigaciones Científicas (*n* = 29; 8.10%) and the University of Queensland (*n* = 22; 6.15%). These publication patterns reflect a combination of biodiversity, funding, and institutional research capacity. The United States’ leadership is sustained by major investments in biomedical and ecological research [22, 23]. Brazil’s prominence reflects both exceptional biodiversity and long‐standing institutional commitment to venom studies, particularly in *Bothrops* and *Crotalus* species [23, 24]. Australia’s contributions stem from its unique, highly venomous fauna and strong translational research programs in toxinology, supported by advanced proteomics and transcriptomics platforms [25–28].

**TABLE 1:** Countries with the highest number of documents on snake venom and transcriptomics from 2002 to 2024.

No.	Country	Number of documents (%)
1	United States	116 (32.40)
2	Brazil	78 (21.79)
3	Australia	50 (13.97)
3	United Kingdom	50 (13.97)
4	China	37 (10.34)
5	Spain	33 (9.22)
6	France	23 (6.42)
7	Costa Rica	21 (5.87)
8	Singapore	18 (5.03)
8	India	18 (5.03)

*Note:* Countries with the same number of documents are assigned the same rank (ties).

**TABLE 2:** The top 10 most productive institutions in snake venom and transcriptomics research from 2002 to 2024.

No.	Institutions	Country	Number of documents (%)
1	Instituto Butantan	Brazil	49 (13.69)
2	Florida State University	United States	32 (8.94)
2	Liverpool School of Tropical Medicine	United Kingdom	32 (8.94)
3	Consejo Superior de Investigaciones Científicas	Spain	29 (8.10)
4	Universidade de São Paulo	Brazil	26 (7.26)
5	CSIC—Instituto de Biomedicina de Valencia (IBV)	Spain	25 (6.98)
6	The University of Queensland	Australia	22 (6.15)
7	Universidad de Costa Rica	Costa Rica	20 (5.59)
8	National University of Singapore	Singapore	18 (5.03)
8	Clemson University	United States	18 (5.03)

*Note:* Institutions with the same number of documents are assigned the same rank (ties).

### 2.3. Keyword Co‐Occurrence and Thematic Structure

A VOSviewer co‐occurrence analysis of keywords from the 358 documents identified 4449 unique terms, with 42 appearing at least 50 times (Figure 2). The network visualization (Figure 2(a)) revealed interconnected clusters centered around “transcriptomics,” “snake venom,” and “proteomics,” illustrating the thematic structure of the field. Overlay visualization (Figure 2(b)) showed a temporal progression: early terms (e.g., “venom gland,” “toxins”) appeared in purple, while recent keywords (e.g., “next‐generation sequencing,” “biomedical applications”) appeared in yellow, reflecting the evolution from foundational research toward applied and high‐throughput studies. However, thematic trends also reveal blind spots: regulatory keywords (e.g., “microRNA,” “lncRNA,” and “epigenetics”) remain underrepresented, and ecological terms (e.g., “prey diversity” and “selection pressure”) are virtually absent.

FIGURE 2(a) Network visualization of co‐occurring terms in snake venom and transcriptomics research. (b) Overlay visualization showing the temporal progression of term usage. A total of 4449 keywords were identified, with 42 terms occurring at least 50 times. Terms in purple appeared earlier in the timeline, while those in yellow emerged later.

**Figure:**
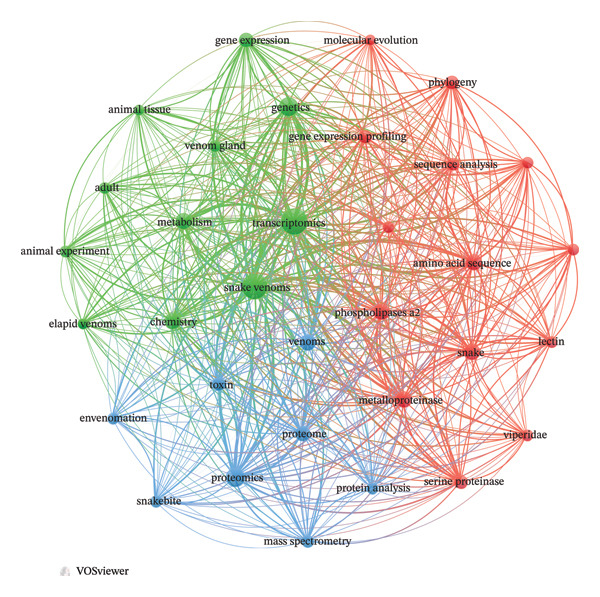
(a)

**Figure:**
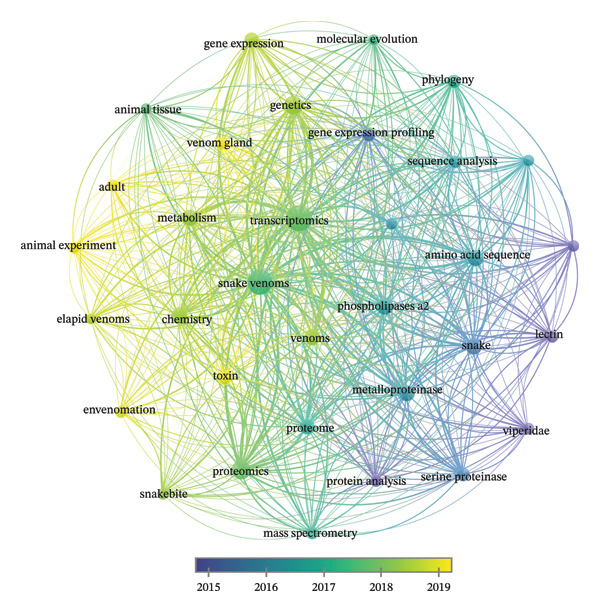
(b)

### 2.4. Taxonomic Coverage of Snake Venom Transcriptomics

To connect the bibliometric landscape with the biological focus of the field, we summarized the snake taxa explicitly represented in the Scopus records by screening titles, abstracts, and keywords for family‐ and genus‐level names (one count per paper per taxon to avoid within‐paper duplication) (Table 3). Across the 358 publications, taxonomic emphasis was strongly skewed toward medically important lineages. At the family level, Elapidae (*n* = 80; 22.35%), Viperidae (*n* = 76; 21.23%), and Colubridae (*n* = 28; 7.82%) were the most frequently mentioned families (Table 3(A)). At the genus level, the literature was dominated by New World viperids and major elapid genera, led by *Bothrops* (*n* = 45; 12.57%), *Crotalus* (*n* = 43; 12.01%), and *Naja* (*n* = 30; 8.38%), followed by *Bungarus, Micrurus, Vipera,* and *Daboia* (Table 3(B)). Overall, 218/358 records (60.89%) contained at least one identifiable snake genus in these bibliographic text fields, indicating that many papers either generalize findings without naming taxa in the indexed text or focus on methodological aspects where species names are not consistently captured in titles/abstracts/keywords. Taken together, these results highlight that venom transcriptomics remains concentrated in a subset of well‐studied, clinically relevant taxa, reinforcing the need for transcriptomics‐driven frameworks that are generalizable beyond a limited set of model venomous snakes.

**TABLE 3:** Taxonomic coverage of snake venom transcriptomics (Scopus, 2002–2024; *n* = 358).

No.	Family	Number of documents (%)
*A. Family-level terms mentioned in title/abstract/keywords*		
1	Elapidae	80 (22.35)
2	Viperidae	76 (21.23)
3	Colubridae	28 (7.82)

**No.**	**Genus**	**Number of documents (%)**

*B. Top genera mentioned in title/abstract/keywords*		
1	*Bothrops*	45 (12.57)
2	*Crotalus*	43 (12.01)
3	*Naja*	30 (8.38)
4	*Bungarus*	18 (5.03)
5	*Micrurus*	15 (4.19)
6	*Vipera*	13 (3.63)
6	*Daboia*	13 (3.63)
8	*Protobothrops*	12 (3.35)
9	*Agkistrodon*	11 (3.07)
9	*Echis*	11 (3.07)
9	*Ophiophagus*	11 (3.07)
10	*Deinagkistrodon*	10 (2.79)
11	*Gloydius*	8 (2.23)
11	*Trimeresurus*	8 (2.23)
11	*Hydrophis*	8 (2.23)

*Note:* Taxa were identified by rule‐based matching of family/genus names in Scopus‐exported Title/Abstract/Author Keywords/Index Keywords fields. Counts reflect the number of papers mentioning a given taxon at least once (i.e., one hit per paper per taxon). In total, 218/358 papers (60.89%) contained at least one identifiable genus in these bibliographic text fields. Genera with the same number of documents are assigned the same rank (ties).

### 2.5. Coverage of the Three Transcriptomic Axes in the Literature

To quantify how the field distributes across the three transcriptomic axes proposed in Section 3, we classified each of the 358 publications using a rule‐based keyword tagging of Scopus‐exported title, abstract, and keyword fields (Table 4). Overall, the literature most frequently addresses Axis 1 (gene family expansion/toxin repertoire and paralog diversity), which was detected in 263 papers (73.46%), reflecting the dominant use of venom gland transcriptomics for toxin discovery, cataloging, and comparative profiling. Axis 2 (tissue‐specific regulatory complexity) was also common (223 papers; 62.29%), but these studies were more heterogeneous, spanning differential expression, isoform‐level variation, and posttranscriptional regulation (e.g., noncoding RNAs). In contrast, Axis 3 (ecological selection pressures) was least represented (147 papers; 41.06%), indicating that comparatively fewer transcriptomic studies explicitly integrate ecological variables such as diet, geography, prey specialization, or ontogeny into their study design (Table 4(A)).

**TABLE 4:** Coverage of the three transcriptomic axes across the literature (Scopus, 2002–2024; *n* = 358).

No.	Transcriptomic axis	Number of documents (%)
*A. Axis-level coverage (multilabel tagging)*		
1.	Axis 1: Gene family expansion (toxin repertoires/paralogs)	263 (73.46)
2.	Axis 2: Tissue‐specific regulatory complexity	223 (62.29)
3.	Axis 3: Ecological selection pressures	147 (41.06)

**No.**	**Axis overlap category**	**Number of documents (%)**

*B. Degree of overlap among axes.*		
1	All three axes (Axis 1 + Axis 2 + Axis 3)	80 (22.35)
2	Two axes (any combination)	144 (40.22)
3	One axis only	105 (29.33)
4	None (no axis keywords detected)	29 (8.10)

*Note:* Axis coverage was determined by rule‐based keyword matching in Scopus‐exported Title/Abstract/Author Keywords/Index Keywords fields. Tagging was multilabel, meaning a single paper could be counted under more than one axis; therefore, axis totals in Panel A are not expected to sum to 358.

Importantly, many studies touched on multiple axes: 80 papers (22.35%) addressed all three axes, and 144 papers (40.22%) addressed two axes, whereas 105 papers (29.33%) were tagged to only one axis (Table 4(B)). A small subset (29 papers; 8.10%) did not match any axis keywords in bibliographic text fields, consistent with papers that describe transcriptomic resources or general venom work without explicitly using the axis‐associated terminology. Collectively, this distribution highlights a strong thematic emphasis on toxin repertoire characterization (Axis 1), with clear opportunities to expand transcriptomics‐driven regulatory (Axis 2) and ecology‐linked (Axis 3) investigations.

In summary, this quantitative overview reveals rapid growth but uneven thematic coverage in venom transcriptomics. The combined evidence from publication trends, keyword structure, taxonomic concentration, and axis coverage motivates the need for a structured framework that links transcriptome‐derived evidence to evolutionary mechanisms, as developed in Section 3.

## 3. A Conceptual Framework for Transcriptomic Evaluation of Venom Evolution

**FIGURE 3:**
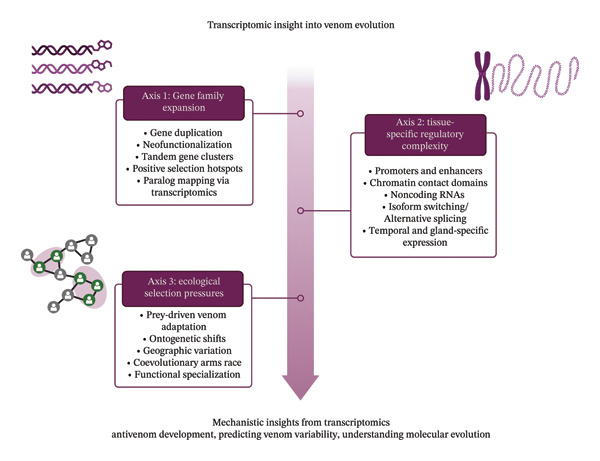
Conceptual framework for evaluating venom evolution using transcriptomic data. The model integrates three axes (gene family expansion, regulatory complexity, and ecological selection pressures) to explain the molecular and functional diversification of snake venom. Each axis is informed by transcriptomic insights and contributes to venom adaptation. The figure was created in BioRender.

Understanding the evolution of snake venom through transcriptomic approaches requires a structured model that accounts for the complex interplay between genomic architecture, regulatory mechanisms, and ecological drivers. Based on the synthesis of current literature and gaps identified in this review, we propose a conceptual framework that integrates three core axes to evaluate venom evolution via transcriptomics: (1) gene family expansion, (2) tissue‐specific regulatory complexity, and (3) ecological selection pressures (Figure 3).

### 3.1. Axis 1: Gene Family Expansion

This axis refers to the duplication, diversification, and neofunctionalization of toxin genes, which are frequently observed across snake lineages. Transcriptomic data can reveal the extent of such expansions, highlighting lineage‐specific patterns and molecular innovations that drive toxin diversity. High‐resolution transcriptomes allow for detailed mapping of paralogous gene clusters, enabling insights into evolutionary pressures acting on different toxin families.

### 3.2. Axis 2: Tissue‐Specific Regulatory Complexity

This axis encompasses the transcriptional and posttranscriptional mechanisms that control venom gene expression within the venom gland. This includes the activity of promoters, enhancers, chromatin organization, and noncoding RNAs. Differences in expression levels, isoform diversity, and temporal regulation can influence the proteomic output of venom, independent of gene copy number. By focusing on this axis, transcriptomic studies can move beyond gene presence to investigate how regulatory dynamics shape venom profiles.

### 3.3. Axis 3: Ecological Selection Pressures

This axis captures the influence of diet, habitat, and predator–prey interactions on venom composition. Transcriptomic variation driven by ecological factors, such as prey specialization, ontogenetic shifts, or geographic isolation, can provide insights into adaptive responses. Comparative transcriptomics across populations or sympatric species can help elucidate how selection shapes venom expression at both the gene and pathway levels.

Figure 3 illustrates how the proposed framework can uncover both shared evolutionary mechanisms (e.g., gene duplication as a diversification engine) and lineage‐specific adaptations (e.g., regulatory plasticity or ecological targeting). It moves the interpretation of transcriptomic data beyond taxonomic inventories toward mechanistic insights into venom evolution. Such multidimensional evaluations may also guide region‐specific antivenom development by clarifying how local ecological or developmental contexts influence venom gene expression profiles. Ultimately, the framework encourages future research to integrate multiomics and ecological data in a hypothesis‐driven manner, fostering a more predictive and functional understanding of venom evolution.

## 4. Gene Family Expansion

To understand how venom composition becomes increasingly diverse and specialized, it is important to connect gene family expansion to what is measurable in venom gland transcriptomes. In transcriptomic datasets, expansion is not inferred solely from genomic copy number, but from (i) the number of expressed paralogs within a toxin family, (ii) the degree of expression dominance (a small subset of paralogs accounts for most toxin transcripts), and (iii) the presence of isoform diversity generated by alternative splicing and transcript variation. Collectively, these patterns provide a practical transcriptomics‐based view of how duplication and divergence translate into functional venom phenotypes through regulatory divergence at the transcriptional and isoform levels.

### 4.1. Snake Venom Metalloproteinases (SVMP), Phospholipases A2 (PLA2), and Snake Venom Serine Proteinase (SVSP): Transcriptomic Signatures

Venom gland transcriptomes consistently show that a few toxin superfamilies dominate expression, yet within each superfamily, there are often multiple expressed paralogs, reflecting the evolutionary history of gene duplication coupled with lineage‐specific retention [29–31]. Importantly, transcriptomic profiling frequently reveals highly uneven expression distributions, where a small number of paralogs are strongly upregulated and likely drive much of the venom phenotype, while many additional paralogs are expressed at lower levels [30, 32, 33]. Taken together, this pattern is compatible with models in which duplication supplies genetic templates, while subsequent divergence occurs primarily through changes in expression regulation rather than extensive coding‐sequence innovation [34–36].

From a transcriptomics perspective, SVMP, PLA2, and SVSP families are best discussed not by domain architecture, but by how their transcripts behave in venom glands [13, 37, 38]. Accordingly, venom gland transcriptomes can resolve (i) family‐level abundance (relative contribution of each toxin family to total venom transcripts), (ii) paralog composition (how many distinct SVMP/PLA2/SVSP transcripts are expressed), and (iii) paralog‐specific expression bias (which transcripts are most highly expressed) [35, 38, 39]. These metrics are directly comparable across species and populations, enabling transcriptome‐driven hypotheses about prey adaptation and ecological specialization.

### 4.2. Isoform Diversification

Beyond paralog number and abundance, transcriptomics reveals toxin diversity through isoform variation, including alternative splicing and transcript processing that can generate functionally distinct toxin variants from the same genomic locus [40, 41]. Venom gland studies have reported multiple toxin isoforms within serine proteases and other families, indicating that transcript‐level diversification can contribute to venom complexity even when genomic copy number is unchanged [40, 41]. Thus, in this framework, “diversification” is not only a product of duplication but also of transcript regulation, where different isoforms may be expressed at different levels across lineages, environments, or life stages [35, 42].

### 4.3. Differential Expression Across Contexts

A key advantage of venom gland transcriptomics is the ability to quantify differential expression of toxin transcripts across species, populations, or ecological conditions (e.g., diet shifts, habitat differences, and ontogeny) [43–45]. Such comparisons commonly show that venom evolution is driven by expression remodeling: Closely related species may share similar toxin families yet differ sharply in which paralogs are upregulated, producing distinct venom phenotypes [35, 46]. This aligns with the concept that selection can act strongly on regulatory variation, favoring increased expression of specific toxin transcripts that improve prey capture or defense [34, 47]. In parallel, transcriptomic data enable “expression specialization” analyses, where paralogs within the same family exhibit distinct expression patterns, suggesting subfunctionalization or neofunctionalization at the regulatory level [34, 42]. These approaches are particularly informative when paired with consistent annotation workflows (orthology clustering, toxin family classification, and isoform‐aware quantification) because they distinguish genuine biological shifts from methodological artifacts.

### 4.4. Recommended Transcriptomics Workflows

To keep analyses reproducible and focused on transcriptomic evidence, studies of gene family expansion should report: (i) venom gland sampling design (species, population, sex, and life stage), (ii) assembly/annotation strategy (including toxin family classification and orthology inference), (iii) isoform‐aware quantification, and (iv) statistical frameworks for differential expression. Where feasible, transcriptomics‐based expansion signals (expressed paralog counts, expression dominance, and isoform diversity) should be integrated with proteomic validation; nevertheless, the transcriptome remains the primary dataset for identifying which toxin genes are actively transcribed and how expression is distributed across paralog repertoires. In summary, gene family expansion in snake venom evolution is most informatively discussed through transcriptomics as an interplay between expressed paralog diversity and expression regulation.

## 5. Regulatory Complexity

While gene duplication provides the raw material for toxin diversity, it is the regulatory architecture that determines when, where, and how these genes are expressed. This section examines the second axis of regulatory complexity, focusing on transcriptional and epigenetic mechanisms that control venom gene expression across different tissues and developmental stages.

### 5.1. Gene Regulation and Expression Patterns

Regulation of gene expression, encompassing promoter regions and epigenetic mechanisms, is fundamental to the adaptive evolution of snake venom. The genome analysis of the king cobra has shown that venom toxin genes have diversified through unique genomic processes such as gene duplication and positive selection, governed by core genetic elements that originate from pancreatic regulatory pathways [8]. Further support for the co‐option of regulatory components is found in studies on the prairie rattlesnake, where extracellular signal‐regulated kinase and unfolded protein response pathways contribute trans‐regulatory factors that control venom gene expression, creating significant cellular heterogeneity [34, 48]. The expression of venom genes is also regulated through conserved cis‐regulatory sequences, including enhancers and promoters, which have persisted over millions of years, underscoring their role in the evolution of venom’s regulatory framework [34]. In the species *Pseudonaja textilis*, the tissue‐specific expression of toxin genes is directed by transcriptional control through cis‐elements and transcription factors, with AG‐rich motifs notably involved in gene silencing [49]. Proteomics analyses in *Sistrurus* rattlesnakes reveal that gene regulation influences venom protein levels significantly, with considerable interindividual variability in protein abundance, highlighting gene regulation as a pivotal factor in venom diversity [50]. The presence of venom gene homologs across various python tissues suggests that venom genes originated from genes with specific expression patterns, facilitating their integration into the specialized venom production system [51]. Additionally, chromatin architecture directs precise expression of venom gene families, where specific chromatin contact domains and transcription factor activity are essential for venom production and regulation [52]. Diverse evolutionary trajectories in venom expression across snake species further exemplify the role of gene expression regulation in local adaptation and phenotypic variation [45]. In pit vipers, adaptive responses to diet involve both protein sequence variation and differential gene expression, with diversity in both domains contributing to venom complexity and efficacy in prey capture [47]. Finally, the physiological demands and signaling mechanisms linked to venom synthesis, including stress‐response pathways and cellular functions, reflect the intricate regulation underlying venom production [53].

In addition to transcriptional and chromatin‐mediated regulation, posttranscriptional mechanisms involving noncoding RNAs have recently been recognized as important modulators of venom gene expression. MicroRNAs (miRNAs) play a crucial role in fine‐tuning toxin transcript abundance in snakes by targeting mRNAs encoding phospholipases, metalloproteinases, and neurotoxins to modulate their stability and translation efficiency [49, 54, 55]. Recent transcriptomic and miRNA‐profiling studies have shown that miRNAs are differentially expressed in venom glands depending on physiological state (such as after venom extraction) and are predicted to regulate a diverse array of toxin transcripts, including those for phospholipases and metalloproteinases [49, 54]. In elapid snakes, miRNAs target a broad spectrum of toxin mRNAs, while in viperids, their regulatory activity appears more focused on metalloproteinase transcripts [54]. Additionally, comparative analyses suggest that posttranscriptional modulation by miRNAs contributes to differences in toxin abundance and composition, highlighting their importance in the dynamic regulation of venom gene expression [54, 55]. Noncoding RNAs, including long noncoding RNAs (lncRNAs) and small interfering RNAs (siRNAs), play a crucial role in orchestrating the temporal and cell‐type‐specific expression of snake venom genes by regulating gene activity at multiple levels [8, 54, 56]. Additionally, lncRNAs have been identified in snake genomes and are implicated in generating venom gene diversity and regulating the expression of myotoxins, further supporting their role in venom system evolution and adaptation [56]. These noncoding RNAs interact with gene promoters, transcription factors, and chromatin structure, enabling dynamic and tissue‐specific control over venom gene expression, which is essential for rapid venom replenishment and adaptation to ecological pressures [8, 16, 54, 56].

## 6. Ecological Selection Pressures

The final axis in our model focuses on the ecological and evolutionary forces that shape venom composition through changes in toxin gene expression and sequence evolution. To keep this section explicitly anchored in transcriptomics, we emphasize how ecological variables (diet, habitat, prey community, and predator–prey dynamics) are investigated using venom gland RNA‐seq, including differential expression, isoform‐level shifts, and comparative transcriptomic analyses across populations, life stages, and species.

### 6.1. Adaptive Significance of Toxin Variability

Ecological pressures such as prey diversity and habitat can drive venom diversification by favoring shifts in which toxin transcripts are highly expressed. In this context, transcriptomic comparisons allow direct testing of ecological hypotheses by quantifying changes in toxin family composition at the mRNA level and identifying specific paralogs that dominate expression under different ecological regimes. For example, diet breadth and prey specialization have been associated with toxin variability across snakes, with generalist feeders often exhibiting broader toxicological repertoires than specialists [57, 58]. In *Spilotes sulphureus*, prey diversity is linked to the evolution of multiple toxins with distinct prey‐specific activities [3], a pattern that can be investigated transcriptomically by asking whether prey‐associated venom phenotypes correspond to shifts in toxin transcript abundance, paralog usage, or isoform profiles. Similarly, differences among sea snakes in venom composition associated with prey preferences and the prey‐specialized predatory ecology of the King Cobra motivate transcriptome‐based comparisons to assess whether ecological specialization corresponds to expression remodeling of particular toxin families or specific toxin transcripts [59, 60]. Dietary shifts in *Echis* and their association with venom toxicity changes likewise provide a conceptual framework for transcriptomic studies that quantify expression changes linked to prey transitions [1]. Importantly, ecological niche shifts can also coincide with altered evolutionary tempo, as suggested by reduced venom complexity and slowed toxin evolution in egg‐eating lineages such as *Aipysurus eydouxii* [61]. In this context, transcriptomics can test whether this corresponds to reduced toxin transcript diversity, reduced expression of canonical toxin families, or altered regulatory states.

### 6.2. Selective Pressures and Molecular Evolution

Ecological selection pressures are ultimately realized through molecular evolution, and venom research provides substantial evidence that positive selection and gene family diversification contribute to toxin innovation. A common model is that gene duplication creates paralog diversity, followed by functional divergence driven by selection, as described for PLA2 gene families that exhibit signatures of positive Darwinian selection and neofunctionalization [62]. In pit vipers, venom proteins can show both strong positive selection and relaxed constraints, supporting rapid molecular evolution that may accompany ecological diversification [63]. The king cobra genome further supports this paradigm, reporting toxin gene expansion via duplication coupled with positive selection consistent with coevolutionary predator–prey dynamics [8]. Broadly, genomic‐era syntheses similarly emphasize duplication and positive selection as key mechanisms in venom protein neofunctionalization and diversification [64].

Although these signatures are often identified using coding‐sequence analyses, transcriptomics adds an essential layer by revealing which genes under selection are actually expressed in venom glands, at what levels, and in which contexts. This is particularly relevant when selection correlates with expression dominance: Studies suggest that highly abundant venom components can experience accelerated evolution, consistent with selection optimizing functional performance and energetic investment in venom production [65]. Positive selection has also been invoked for toxin families beyond PLA2, including SVMPs, where domain modification and selection on exposed residues may contribute to functional change [66], and cysteine‐rich secretory proteins (CRISPs), where accelerated evolution and directed mutations are proposed to enhance biological targeting [67]. Gene family expansion and adaptive evolution in rattlesnakes further illustrate how duplication and selection can contribute to novel biochemical capabilities [68]. Finally, evidence of reciprocal selection in prey resistance (e.g., convergent resistance to neurotoxins) reinforces an evolutionary arms race framework that is central to interpreting venom adaptation [69].

Taken together, positioning molecular evolution here clarifies the narrative: Ecological pressures provide the selective context, molecular evolution provides the mechanistic explanation, and transcriptomics provides the critical readout of expressed toxin repertoires and regulatory remodeling that translate selection into venom phenotypes.

## 7. Case Studies of Transcriptomic Insights in Evolution

This section applies the three conceptual axes (gene family expansion, regulatory complexity, and ecological selection pressures) to case studies in *Naja, Bothrops,* and *Trimeresurus*, demonstrating how each axis contributes to venom diversity and specialization (Table 5).

**TABLE 5:** Integrated transcriptomic insights into venom evolution in *Naja, Bothrops*, and *Trimeresurus* using the three‐axis model.

Snake genus/species	Gene family expansion (Axis 1)	Regulatory complexity (Axis 2)	Ecological selection pressures (Axis 3)	References
*Naja kaouthia* (Malaysia vs. Thailand)	High redundancy in 23 gene families; 3FTx dominance	Geographic variation in transcript upregulation (α‐neurotoxins vs. short neurotoxins)	Regional prey differences; spitting behavior possibly influencing selection	[15]
*N. atra*	Dominance of 3FTx (95.8% of transcriptome); tandem duplications	Rapid diversification in both major and minor toxin families	Adaptation across East Asia; prey‐specific toxin variation	[70]
*N. sumatrana*	21 toxin families identified; dominance of 3FTx	Genetic diversity via de novo transcriptomics	Adaptation to equatorial environments; defensive spitting	[71]
*N. kaouthia* (China)	28 toxin families (transcriptome); 6 families (proteome)	Poor antivenom binding suggests unique regulatory outcomes	Selection divergence from reference species; prey/predator mismatch	[72]
*N. naja*	139 toxin genes across 33 families from genome and transcriptome	High‐resolution expression data support recombinant antivenom design	Ecological generalist with broad venom target range	[74]
*N. oxiana*, *N. naja* (Pakistan)	High venom proteome diversity; presence of extracellular vesicles	Posttranslational modifications (e.g., N‐terminal acetylation)	Enhanced adaptation to regional prey and defense needs	[73]
*Bothrops jararaca*	82 full‐length toxin genes identified	Toxin genes co‐expressed in other tissues, e.g., pancreas	Complex prey–predator interactions in tropical forests	[75]
*B. cotiara* and *B. fonsecai*	Shared toxin isoforms across species	Differential expression of PLA2, SVMP, and SVSP genes	Distinct ecotypes suggest fine‐scale ecological adaptation	[35]
*B. alternatus*	Predominance of SVMPs, bradykinin‐potentiating peptides	Absence of basic PLA2 linked to reduced myotoxicity	Venom composition reflects ecological roles of prey capture	[76]
*B. jararacussu* (ontogeny)	125 putative toxin transcripts	Developmental shifts: more PLA2 in adults, more SVMP in juveniles	Ontogenetic adaptation to prey size and availability	[77]
*B. jararaca* (sex and age)	Dynamic proteomic rearrangement with development	Sex‐ and age‐linked changes in toxin expression patterns	Life stage and gender influence prey selection and venom needs	[78]
*Trimeresurus gracilis*	155 proteoforms across 13 toxin families	Differential proteoform expression among shared toxin types	Ecological divergence with *Ovophis okinavensis*	[79]
*T. flavoviridis*	Accelerated evolution of PLA2 gene family	Exon‐specific substitution rates reflect positive selection	Regional adaptation driven by prey ecology	[80]
*T. flavoviridis* (Okinawa vs. other islands)	Silencing of [Lys49] PLA2 genes in Okinawa populations	Formation of pseudogenes implies transcriptional suppression	Island biogeography shapes venom gene retention/loss	[81]
*T. gramineus* and *T. flavoviridis*	Divergence in protein‐coding regions of PLA2 genes	Noncoding regions conserved, coding regions rapidly evolving	Evolution shaped by prey‐specific ecological constraints	[82]
*T. stejnegeri*	Discovery of novel PLA2 isoforms from different regions	Multiple ancestral lineages; geographic variation in transcriptome	Geographic isolation drives toxin diversification	[83]

### 7.1. *Naja* Species

Evolutionary insights gleaned from transcriptomic studies have greatly advanced our understanding of venom evolution in *Naja* species. For example, a comparative transcriptomic analysis of venom glands in *N*. *kaouthia* from Malaysia and Thailand shed light on geographical variations in venom composition and the presence of novel sequences. This study demonstrated high redundancy among toxin transcripts, covering 23 gene families, with a predominance of three‐finger toxins (3FTxs). The distinct expression patterns of 3FTxs between the geographical populations suggested either upregulation of specific primary toxins or enhanced degradation of certain transcripts. In particular, the upregulation of α‐neurotoxins in Thai specimens contrasted with the upregulation of short neurotoxin isoforms in Malaysian specimens, underscoring geographical variability in venom composition and toxicity [15].

In another study by Jiang et al., transcriptomic analyses of venom glands from *N*. *atra* provided valuable insights into toxin gene evolution. The 3FTxs constituted the dominant toxins in *N. atra*, representing 95.8% of the venom transcriptome, and the study underscored the role of tandem duplications in expanding multigene toxin families. This research also suggested that both toxin multigene families and less abundant toxins may have undergone rapid diversifying evolution [70]. Additionally, de novo transcriptomics of the venom glands of *N*. *sumatrana*, the equatorial spitting cobra, identified 21 toxin families, with 3FTxs as the most prevalent. This diversity elucidates the pathophysiology of *N. sumatrana* envenomation and highlights the extensive genetic diversity present in cobra venom toxins [71].

Moreover, a comprehensive omics study on *N*. *kaouthia* from China identified six protein families in the venom proteome and 28 in the venom gland transcriptome. This study noted the dominance of 3FTx and PLA2 families and reported that several venom components could not be effectively immunocaptured by available *N*. *atra* antivenom, suggesting a need for new antivenom formulations [72]. Proteomic analyses of *N*. *naja* and *N*. *oxiana* from Pakistan further revealed venom complexity and posttranslational modifications, including N‐terminal acetylation, which may provide insight into venom protein regulation. The study also identified a highly diverse proteome and extracellular vesicles within the venom, findings that could inform specific antivenom production and enhance understanding of envenomation mechanisms [73].

Finally, the reference genome and transcriptome of the *N. naja* enabled a comprehensive characterization of venom toxins, identifying 139 toxin genes spanning 33 toxin families. This high‐quality genome assembly supports the development of effective humanized recombinant antivenoms, alongside facilitating evolutionary research and venom‐based drug discovery [74]. Collectively, these studies enhance our knowledge of genetic diversity, evolutionary processes, and functional roles of venom components in *Naja* species, underscoring the significance of transcriptomic data in venom evolution research.

### 7.2. Bothrops Species

Case studies exploring transcriptomic insights have provided profound revelations into venom evolution in *Bothrops* species. For example, a detailed transcriptomic analysis of *Bothrops jararaca* highlighted the intricacies of venom evolution by identifying 82 full‐length toxin genes in the venom gland and additional products from other tissues, such as pancreatic enzymes. This study revealed that around 20% of toxin genes are coexpressed at low levels in other body tissues and pinpointed the closest paralogs to toxin genes across eight toxin classes, illustrating the complex evolutionary and production pathways of venom in snakes [75]. In another investigation, the venom gland transcriptomes of *B*. *cotiara* and *B*. *fonsecai* were analyzed to elucidate the regulatory mechanisms underlying their unique venom compositions. The research identified similar toxin isoforms in both species but revealed distinct expression profiles for PLA2, certain SVMPs, and SVSPs, suggesting that gene regulation plays a pivotal role in venom evolution, especially in species with similar ecotypes [35]. Additionally, a transcriptomic analysis of venom gland genes in *B*. *alternatus* identified major toxin groups, including metalloproteinases, bradykinin‐potentiating peptides, phospholipases A2, serine proteinases, and C‐type lectins. The prevalence of type PIII metalloproteinases was linked to hemorrhagic activity in the venom, whereas the absence of basic PLA2 was associated with reduced myotoxicity compared with other *Bothrops* species [76]. Furthermore, research on the venom gland transcripts of *B*. *jararacussu* across varying specimen sizes revealed ontogenetic shifts in venom composition. The study identified 125 distinct putative toxin transcripts, with basic myotoxic phospholipases A2 more abundant in larger snakes, while PIII‐class SVMPs dominated in smaller snakes. These findings suggest that transcriptional events significantly contribute to venom variability, enhancing predatory efficacy in younger, smaller snakes [77]. Finally, a study on the venom gland proteome of *B*. *jararaca* across developmental stages and genders demonstrated dynamic rearrangements in the proteomic landscape. Variations in the expression of cellular proteins were shown to influence toxin levels, indicating that ecological traits and shifts in protein synthesis support the species’ adaptive capacity for targeting different prey types over its lifespan [78]. Collectively, these studies highlight the complexity and diversity of venom evolution in *Bothrops* species, driven by gene regulation, ontogenetic shifts, and ecological adaptations.

### 7.3. *Trimeresurus* Species

Investigations into transcriptomic insights on venom evolution have yielded substantial findings in *Trimeresurus* species. For example, the venom proteomics of *Trimeresurus gracilis*, a pit viper endemic to Taiwan, has been thoroughly examined. Proteomic studies identified 155 toxin proteoforms across 13 viperid venom toxin families, with a notable abundance of SVMPs and serine proteases. Structural similarities of PI‐SVMP, kallikrein‐like proteases, CRISPs, and VEGF‐F between *T. gracilis* and *Ovophis okinavensis* were also observed, suggesting a close phylogenetic relationship, albeit with varying expression levels likely shaped by ecological and prey‐specific factors [79]. In another study, accelerated evolution of venom gland PLA2 isozymes in *T*. *flavoviridis* was explored, revealing that protein‐coding regions of these genes evolved at higher substitution rates than introns, indicative of Darwinian accelerated evolution. This points to the rapid evolution of PLA2 types within venom, contributing to their diverse physiological activities [80]. Additionally, the geographic evolution of venom gland PLA2 isoenzymes in *T. flavoviridis* from different islands showed notable regional differences. For instance, Okinawa populations lack myotoxic [Lys49]PLA2 isoenzymes present in the Amami‐Oshima and Tokunoshima populations, implying these genes have been silenced to form pseudogenes, likely as an adaptation to local environmental conditions [81]. Further evidence of accelerated evolution was observed in protein‐coding regions of PLA2 isozyme genes in crotaline venom glands, including *T*. *gramineus* and *T. flavoviridis*, where rapid evolution was restricted to protein‐coding regions, with noncoding regions remaining highly conserved [82]. Moreover, molecular analyses of PLA2 venom isoforms in *T*. *stejnegeri* from various locations in Taiwan and China demonstrated significant geographic variation and multiple ancestral lineages, with novel PLA2 isoforms identified. This suggests that geographic isolation and prey diversity contribute to venom variation [83]. Collectively, these studies highlight the intricate evolutionary dynamics shaping venom components in *Trimeresurus* species, driven by ecological pressures and genetic adaptations. Insights from transcriptomic and proteomic analyses deepen our understanding of the molecular mechanisms driving venom evolution in these pit vipers.

## 8. Future Perspectives: Emerging Directions and Technological Advances

### 8.1. Integration With Other Omics Approaches

The complexity of venom composition and its evolutionary dynamics warrants a multidimensional analytical approach, combining transcriptomics with genomics, proteomics, and metabolomics. This integrative omics framework allows for a holistic characterization of venom profiles and a comprehensive understanding of the genetic and biochemical underpinnings of venom evolution. The study of transcriptomics, which examines RNA transcripts generated by the genome, offers an in‐depth understanding of gene expression patterns within venom glands. When combined with genomics, which explores the complete DNA sequence and genome structure, this approach allows researchers to identify the genetic foundations of venom synthesis and the evolutionary processes that contribute to venom diversity. For example, sequencing the king cobra genome revealed that venom toxin genes evolved through gene duplication and positive selection, underscoring the dynamic evolution of venom genes [8]. Meanwhile, proteomics, focusing on the large‐scale analysis of proteins, complements transcriptomics by identifying and quantifying the protein components in venom. This technique has been applied to elucidate the diversity and prevalence of protein families in snake venoms, exposing considerable differences in venom composition across species [19, 28]. Proteomic studies have demonstrated that viper venoms exhibit greater compositional complexity than elapid venoms, involving a larger array of protein families [28]. Integrating proteomic and transcriptomic data facilitates a more complete understanding of venom composition and the functional significance of various toxins [18]. Together, these approaches create a more complete picture of the functional and adaptive relevance of venom, elucidating how specific genetic variants contribute to ecological fitness. Moving forward, integrating multiomics datasets will not only enhance our understanding of the evolutionary pressures shaping venom systems but also support the development of tailored antivenom strategies that address the specific venom profiles of distinct snake populations.

### 8.2. Advancements in Single‐Cell Transcriptomics

The advent of single‐cell transcriptomics presents a promising advancement for venom research, particularly in the study of venom gland architecture and cell‐specific toxin expression. Traditional bulk transcriptomic approaches often mask cell‐specific expression patterns, limiting insight into the precise cellular contributions to venom composition. Single‐cell RNA sequencing of venom glands from *Crotalus viridis* has revealed a significant degree of variability in venom gene expression across individual cells. This heterogeneity is driven by the recruitment of trans‐regulatory elements from pathways such as the extracellular signal‐regulated kinase and unfolded protein response pathways, which orchestrate the expression of various venom toxins in a phased manner among a population of secretory cells. Such a regulatory framework implies that cellular constraints, including steric limitations on chromatin or stress in the endoplasmic reticulum, may be mitigated through variable expression patterns across cells [48]. Furthermore, the development of venom gland organoids has emerged as a robust model for investigating venom synthesis and cellular diversity in vitro. These organoids, derived from both embryonic and adult venom gland tissues, retain the cellular complexity of the native gland and can be adjusted to control cell type composition by altering media conditions. Single‐cell RNA sequencing of these organoids has identified specific venom‐producing cell types alongside proliferative cells expressing homologs of established mammalian stem cell markers, underscoring the intricate cellular organization of venom glands [84, 85]. Additionally, the capacity to dissociate organoids into individual cells and conduct subsequent assays, including quantitative real‐time PCR and single‐cell RNA sequencing, has enabled comprehensive studies of venom production and cell differentiation. This method not only advances our understanding of venom gland biology but also offers a safer, more sustainable approach to venom production, essential for the advancement of antivenoms and novel therapeutic agents [85]. Single‐cell transcriptomics has the potential to uncover cellular subtypes involved in the synthesis and secretion of distinct toxin classes, thus refining our understanding of how venom is assembled at a cellular level. Additionally, single‐cell analyses could reveal how various venom‐producing cells respond to different environmental or physiological stimuli, providing new perspectives on venom regulation. Such insights are invaluable for exploring the evolutionary origins of venom gland complexity and adaptive changes in gene regulation that have optimized venom efficiency in response to ecological demands.

### 8.3. Computational Modeling of Venom Evolution

Bioinformatics and computational modeling stand as essential tools for advancing our understanding of venom evolution, particularly in the field of predictive evolutionary trends and functional adaptation [21, 86–88]. Computational approaches such as phylogenomic analyses, ancestral state reconstruction, and adaptive evolution modeling can shed light on the historical trajectories of toxin gene families and elucidate selective pressures acting upon them [21, 86, 87, 89]. Machine learning algorithms, in particular, hold promise for predicting evolutionary hotspots within toxin gene regions, enabling the identification of critical amino acid residues associated with enhanced toxicity or novel functionality [21, 86, 90]. Beyond evolutionary inference, computational pipelines are increasingly used to bridge venom “omics” with translational discovery; for example, venom‐derived peptide datasets have been leveraged in in silico screening frameworks to prioritize candidate bioactive peptides with therapeutic potential (e.g., peptides predicted to inhibit butyrylcholinesterase as a target relevant to Alzheimer’s disease) [91]. Similarly, structure‐based virtual screening approaches have been applied to identify phytochemical candidates predicted to inhibit key venom enzymes such as PLA2, offering a complementary computational route to antitoxin discovery [92]. Additionally, computational models that simulate ecological interactions, such as predator–prey dynamics, can further inform the evolutionary context of venom adaptation by revealing how ecological pressures shape venom composition and function [57, 93, 94]. Such models offer predictive insights into how venom profiles may evolve in response to environmental changes, providing a valuable framework for forecasting potential shifts in venom composition across snake populations [57, 94, 95]. As computational power and algorithmic sophistication continue to advance, these models will become increasingly instrumental in dissecting the evolutionary complexities of venom systems, enhancing both our theoretical understanding and practical applications in antivenom development and biodiversity conservation [93–95].

## 9. Conclusion

Transcriptomics has become a cornerstone in understanding snake venom evolution, offering insights into gene family expansion, regulatory complexity, and ecological selection pressures. These three axes provide a unifying framework to interpret how venom diversity arises and adapts across species. While advances in sequencing technologies have accelerated discoveries, challenges remain in standardization, annotation, and equitable access to data and resources. Future progress will require the convergence of multiomics strategies, breakthroughs in single‐cell transcriptomics, and the growing application of computational modeling. Broadening taxonomic coverage and fostering global collaboration will be essential to fully realize the potential of transcriptomic research in venom biology.

## Author Contributions

Fajar Sofyantoro: conceptualization, data curation, formal analysis, funding acquisition, investigation, methodology, visualization, writing–original draft, and writing–review and editing. Wisnu Ananta Kusuma: conceptualization, funding acquisition, formal analysis, validation, and writing–review and editing. Setyanto Tri Wahyudi: formal analysis, validation, and writing–review and editing. Favorisen Rosyking Lumbanraja: formal analysis, validation, and writing–review and editing. Hendra Rahmawan: formal analysis, validation, and writing–review and editing. Heru Cahya Rustamaji: formal analysis, validation, and writing–review and editing. Dhadhang Wahyu Kurniawan: conceptualization, funding acquisition, formal analysis, validation, and writing–review and editing. Undri Rastuti: formal analysis, validation, and writing–review and editing. Wahyu Aristyaning Putri: conceptualization, funding acquisition, formal analysis, validation, and writing–review and editing. Wiko Arif Wibowo: conceptualization, funding acquisition, formal analysis, validation, and writing–review and editing. Dwi Sendi Priyono: conceptualization, funding acquisition, formal analysis, validation, and writing–review and editing. Donan Satria Yudha: conceptualization, formal analysis, validation, and writing–review and editing. Slamet Raharjo: conceptualization, formal analysis, validation, and writing–review and editing. Tri Rini Nuringtyas: conceptualization, formal analysis, funding acquisition, investigation, supervision, writing–original draft, and writing–review and editing.

## Funding

This work was supported by the Directorate of Research, Technology, and Community Service, Ministry of Education, Culture, Research, and Technology, through the Strategic Research Collaboration Program (KATALIS), grant number 013/E5/PG.02.00/PL.BATCH.2/2024.

## Conflicts of Interest

The authors declare no conflicts of interest.

## Supporting Information

Additional supporting information can be found online in the Supporting Information section.

## Supporting information


**Supporting Information** Supporting Table S1 presents the complete list of documents included in the bibliometric analysis of snake venom transcriptomics. The dataset was retrieved from the Scopus database using the search terms “(transcriptome OR transcriptomics) AND (snake AND venom)” within the Article Title, Abstract, and Keywords fields. The table includes the bibliographic records used to analyze publication trends, document types, geographic and institutional contributions, keyword co‐occurrence patterns, taxonomic coverage, and thematic classification according to the three transcriptomic axes proposed in this review.

## Data Availability

The dataset used in the current study will be made available upon request from the corresponding author.

## References

[1] Barlow A. , Pook C. E. , Harrison R. A. , and Wüster W. , Coevolution of Diet and Prey-Specific Venom Activity Supports the Role of Selection in Snake Venom Evolution, Proceedings of the Royal Society A: Mathematical, Physical and Engineering Sciences B. (2009) 276, no. 1666, 2443–2449, 10.1098/rspb.2009.0048, 2-s2.0-66749139721. PMC2690460 19364745

[2] Sofyantoro F. , Septriani N. I. , Yudha D. S. et al., Zebrafish as Versatile Model for Assessing Animal Venoms and Toxins: Current Applications and Future Prospects, Zebrafish. (2024) 21, no. 3, 0088–0242, 10.1089/zeb.2023.0088. 38608228

[3] Modahl C. M. , Mrinalini F. S. , and Mackessy S. P. , Adaptive Evolution of Distinct Prey-Specific Toxin Genes in Rear-Fanged Snake Venom, Proceedings of the Royal Society A: Mathematical, Physical and Engineering Sciences B. (2018) 285, no. 1884, 10.1098/rspb.2018.1003, 2-s2.0-85051805199. PMC6111164 30068680

[4] Mackessy S. P. and Saviola A. J. , Understanding Biological Roles of Venoms Among the Caenophidia: the Importance of Rear-Fanged Snakes, Integrative and Comparative Biology. (2016) 56, no. 5, 1004–1021, 10.1093/icb/icw110, 2-s2.0-84996528044. 27639275

[5] Gibbs H. L. , Sanz L. , Pérez A. et al., The Molecular Basis of Venom Resistance in a Rattlesnake‐Squirrel Predator‐Prey System, Molecular Ecology. (2020) 29, no. 15, 2871–2888, 10.1111/mec.15529. 32593182

[6] Holding M. L. , Biardi J. E. , and Gibbs H. L. , Coevolution of Venom Function and Venom Resistance in a Rattlesnake Predator and Its Squirrel Prey, Proceedings of the Royal Society A: Mathematical, Physical and Engineering Sciences B. (2016) 283, no. 1829, 10.1098/rspb.2015.2841, 2-s2.0-84964703115. PMC4855376 27122552

[7] Robinson K. E. , Holding M. L. , Whitford M. D. , Saviola A. J. , Yates J. R. , and Clark R. W. , Phenotypic and Functional Variation in Venom and Venom Resistance of Two Sympatric Rattlesnakes and Their Prey, Journal of Evolutionary Biology. (2021) 34, no. 9, 1447–1465, 10.1111/jeb.13907. 34322920

[8] Vonk F. J. , Casewell N. R. , Henkel C. V. et al., The King Cobra Genome Reveals Dynamic Gene Evolution and Adaptation in the Snake Venom System, Proceedings of the National Academy of Sciences of the United States of America. (2013) 110, no. 51, 20651–20656, 10.1073/pnas.1314702110, 2-s2.0-84890842764. 24297900 PMC3870661

[9] Kazandjian T. D. , Petras D. , Robinson S. D. et al., Convergent Evolution of Pain-Inducing Defensive Venom Components in Spitting Cobras, Science. (2021) 371, no. 6527, 386–390, 10.1126/science.abb9303. 33479150 PMC7610493

[10] Healy K. , Carbone C. , and Jackson A. L. , Davies J. , Snake Venom Potency and Yield are Associated With Prey-Evolution, Predator Metabolism and Habitat Structure, Ecology Letters. (2019) 22, no. 3, 527–537, 10.1111/ele.13216, 2-s2.0-85059638889. 30616302

[11] Hofmann E. P. , Rautsaw R. M. , Mason A. J. , Strickland J. L. , and Parkinson C. L. , Duvernoy’s Gland Transcriptomics of the Plains Black-Headed Snake, *Tantilla nigriceps* (Squamata, Colubridae): Unearthing the Venom of Small Rear-Fanged Snakes, Toxins. (2021) 13, no. 5, 10.3390/toxins13050336. PMC8148590 34066626

[12] Holding M. L. , Margres M. J. , Mason A. J. , Parkinson C. L. , and Rokyta D. R. , Evaluating the Performance of De Novo Assembly Methods for Venom-Gland Transcriptomics, Toxins. (2018) 10, no. 6, 10.3390/toxins10060249, 2-s2.0-85049099414. PMC6024825 29921759

[13] Tan C. H. , Tan K. Y. , Ng T. S. , Tan N. H. , and Chong H. P. , De Novo Venom Gland Transcriptome Assembly and Characterization for Calloselasma Rhodostoma (Kuhl, 1824), the Malayan Pit Viper From Malaysia: Unravelling Toxin Gene Diversity in a Medically Important Basal Crotaline, Toxins. (2023) 15, no. 5, 10.3390/toxins15050315. PMC10222492 37235350

[14] Aphrodita A. , Sentono D. N. , Yudha D. S. et al., Comparative Analysis of Hemotoxic, Myotoxic, and Inflammatory Profiles of *Calloselasma rhodostoma* and *Trimeresurus insularis* Venoms in Mice, Narrative Journal. (2025) 5, no. 2, 1–16, 10.52225/narraj.v5i2.1874. PMC12425531 40951459

[15] Tan K. Y. , Tan C. H. , Chanhome L. , and Tan N. H. , Comparative Venom Gland Transcriptomics of *Naja kaouthia* (Monocled Cobra) From Malaysia and Thailand: Elucidating Geographical Venom Variation and Insights Into Sequence Novelty, PeerJ. (2017) 5, 10.7717/peerj.3142, 2-s2.0-85017000737. PMC5384570 28392982

[16] Currier R. B. , Calvete J. J. , Sanz L. , Harrison R. A. , Rowley P. D. , and Wagstaff S. C. , Spilianakis C. B. , Unusual Stability of Messenger RNA in Snake Venom Reveals Gene Expression Dynamics of Venom Replenishment, PLoS One. (2012) 7, no. 8, 10.1371/journal.pone.0041888, 2-s2.0-84864697026. PMC3413681 22879897

[17] Sofyantoro F. , Sudaryadi I. , Yudha D. S. , Raharjo S. , Purwestri Y. A. , and Nuringtyas T. R. , Renal Toxicity in Snakebite Envenomation: Insights Into Pathophysiology, Risk Factors, and Management Strategies, Journal of Current Science and Technology. (2025) 15, no. 4, 10.59796/jcst.V15N4.2025.138.

[18] Modahl C. M. , Saviola A. J. , and Mackessy S. P. , Integration of Transcriptomic and Proteomic Approaches for Snake Venom Profiling, Expert Review of Proteomics. (2021) 18, no. 10, 827–834, 10.1080/14789450.2021.1995357. 34663159

[19] Tan C. H. , Snake Venomics: Fundamentals, Recent Updates, and a Look to the next Decade, Toxins. (2022) 14, no. 4, 10.3390/toxins14040247. PMC9028316 35448856

[20] Nachtigall P. G. , Rautsaw R. M. , Ellsworth S. A. et al., Toxcodan: A New Toxin Annotator and Guide to Venom Gland Transcriptomics, Briefings in Bioinformatics. (2021) 22, no. 5, 10.1093/bib/bbab095. 33866357

[21] Roman-Ramos H. and Ho P. L. , Current Technologies in Snake Venom Analysis and Applications, Toxins. (2024) 16, no. 11, 10.3390/toxins16110458. PMC11598588 39591213

[22] Groneberg D. A. , Geier V. , Klingelhöfer D. , Gerber A. , Kuch U. , and Kloft B. , De Silva J. , Snakebite Envenoming—A Combined Density Equalizing Mapping and Scientometric Analysis of the Publication History, PLoS Neglected Tropical Diseases. (2016) 10, no. 11, 10.1371/journal.pntd.0005046, 2-s2.0-85007337286. PMC5098783 27820835

[23] Sofyantoro F. , Yudha D. S. , Lischer K. et al., Bibliometric Analysis of Literature in Snake Venom-Related Research Worldwide (1933–2022), Animals. (2022) 12, no. 16, 10.3390/ani12162058. PMC9405337 36009648

[24] Assis R. A. , Bittar B. B. , Amorim N. P. L. et al., Studies About Snake Peptides: A Review About Brazilian Contribution, Brazilian Archives of Biology and Technology. (2022) 65, 10.1590/1678-4324-2022210421.

[25] Birrell G. W. , Earl S. T. H. , Wallis T. P. et al., The Diversity of Bioactive Proteins in Australian Snake Venoms, Molecular & Cellular Proteomics. (2007) 6, no. 6, 973–986, 10.1074/mcp.M600419-MCP200, 2-s2.0-34347387046. 17317661

[26] Jackson T. , Koludarov I. , Ali S. et al., Rapid Radiations and the Race to Redundancy: An Investigation of the Evolution of Australian Elapid Snake Venoms, Toxins. (2016) 8, no. 11, 10.3390/toxins8110309, 2-s2.0-84993982059. PMC5127106 27792190

[27] Di Fabio J. L. , Cortés Castillo M. D. L. Á. , and Griffiths E. , Landscape of Research, Production, and Regulation in Venoms and Antivenoms: A Bibliometric Analysis, Revista Panamericana de Salud Públic. (2021) 45, 10.26633/RPSP.2021.55. PMC8139637 34035797

[28] Tasoulis T. , Pukala T. L. , and Isbister G. K. , Investigating Toxin Diversity and Abundance in Snake Venom Proteomes, Frontiers in Pharmacology. (2022) 12, 10.3389/fphar.2021.768015. PMC8795951 35095489

[29] Shibata H. , Chijiwa T. , Oda-Ueda N. et al., The Habu Genome Reveals Accelerated Evolution of Venom Protein Genes, Scientific Reports. (2018) 8, no. 1, 10.1038/s41598-018-28749-4, 2-s2.0-85050652583. PMC6062510 30050104

[30] Margres M. J. , Aronow K. , Loyacano J. , and Rokyta D. R. , The Venom-Gland Transcriptome of the Eastern Coral Snake (*Micrurus fulvius*) Reveals High Venom Complexity in the Intragenomic Evolution of Venoms, BMC Genomics. (2013) 14, no. 1, 10.1186/1471-2164-14-531, 2-s2.0-84880901893. PMC3750283 23915248

[31] Rokyta D. R. , Wray K. P. , and Margres M. J. , The Genesis of an Exceptionally Lethal Venom in the Timber Rattlesnake (*Crotalus horridus*) Revealed Through Comparative venom-gland Transcriptomics, BMC Genomics. (2013) 14, no. 1, 10.1186/1471-2164-14-394, 2-s2.0-84878814663. PMC3701607 23758969

[32] Casewell N. R. , Wagstaff S. C. , Wüster W. et al., Medically Important Differences in Snake Venom Composition are Dictated by Distinct Postgenomic Mechanisms, Proceedings of the National Academy of Sciences of the United States of America. (2014) 111, no. 25, 9205–9210, 10.1073/pnas.1405484111, 2-s2.0-84903465784. 24927555 PMC4078820

[33] Cerda P. A. , Crowe-Riddell J. M. , Gonçalves D. J. P. , Larson D. A. , Duda T. F. , and Davis Rabosky A. R. , Divergent Specialization of Simple Venom Gene Profiles Among Rear-Fanged Snake Genera (Helicops and Leptodeira, Dipsadinae, Colubridae), Toxins. (2022) 14, no. 7, 10.3390/toxins14070489. PMC9319703 35878227

[34] Perry B. W. , Gopalan S. S. , Pasquesi G. I. M. et al., Snake Venom Gene Expression is Coordinated by Novel Regulatory Architecture and the Integration of Multiple Co-Opted Vertebrate Pathways, Genome Research. (2022) 32, no. 6, 1058–1073, 10.1101/gr.276251.121. 35649579 PMC9248877

[35] Nachtigall P. G. , Freitas-de-Sousa L. A. , Mason A. J. , Moura-da-Silva A. M. , Grazziotin F. G. , and Junqueira-de-Azevedo I. L. M. , Differences in PLA2 Constitution Distinguish the Venom of Two Endemic Brazilian Mountain Lanceheads, *Bothrops cotiara* and *Bothrops fonsecai* , Toxins. (2022) 14, no. 4, 10.3390/toxins14040237. PMC9028134 35448846

[36] Hargreaves A. D. , Swain M. T. , Hegarty M. J. , Logan D. W. , and Mulley J. F. , Restriction and Recruitment—Gene Duplication and the Origin and Evolution of Snake Venom Toxins, Genome Biology and Evolution. (2014) 6, no. 8, 2088–2095, 10.1093/gbe/evu166, 2-s2.0-84907537182. 25079342 PMC4231632

[37] Casewell N. R. , Harrison R. A. , Wüster W. , and Wagstaff S. C. , Comparative Venom Gland Transcriptome Surveys of the Saw-Scaled Vipers (Viperidae: Echis) Reveal Substantial Intra-Family Gene Diversity and Novel Venom Transcripts, BMC Genomics. (2009) 10, no. 1, 10.1186/1471-2164-10-564, 2-s2.0-71949086303. PMC2790475 19948012

[38] Adisakwattana P. , Chanhome L. , Chaiyabutr N. , Phuphisut O. , Reamtong O. , and Thawornkuno C. , Venom-Gland Transcriptomics of the Malayan Pit Viper (*Calloselasma rhodostoma*) for Identification, Classification, and Characterization of Venom Proteins, Heliyon. (2023) 9, no. 5, 10.1016/j.heliyon.2023.e15476. PMC10160700 37153433

[39] Leonardi A. , Sajevic T. , Pungerčar J. , and Križaj I. , Comprehensive Study of the Proteome and Transcriptome of the Venom of the Most Venomous European Viper: Discovery of a New Subclass of Ancestral Snake Venom Metalloproteinase Precursor-Derived Proteins, Journal of Proteome Research. (2019) 18, no. 5, 2287–2309, 10.1021/acs.jproteome.9b00120, 2-s2.0-85065174347. 31017792 PMC6727599

[40] Ye X. , He C. , Yang Y. et al., Comprehensive Isoform-Level Analysis Reveals the Contribution of Alternative Isoforms to Venom Evolution and Repertoire Diversity, Genome Research. (2023) 33, no. 9, 1554–1567, 10.1101/gr.277707.123. 37798117 PMC10620052

[41] Ogawa T. , Oda-Ueda N. , Hisata K. et al., Alternative mRNA Splicing in Three Venom Families Underlying a Possible Production of Divergent Venom Proteins of the Habu Snake, *Protobothrops flavoviridis* , Toxins. (2019) 11, no. 10, 10.3390/toxins11100581, 2-s2.0-85073118763. PMC6832531 31600994

[42] Amazonas D. R. , Portes-Junior J. A. , Nishiyama-Jr M. Y. et al., Molecular Mechanisms Underlying Intraspecific Variation in Snake Venom, Journal of Proteomics. (2018) 181, 60–72, 10.1016/j.jprot.2018.03.032, 2-s2.0-85044988021. 29621647

[43] Rokyta D. R. , Margres M. J. , Ward M. J. , and Sanchez E. E. , The Genetics of Venom Ontogeny in the Eastern Diamondback Rattlesnake (*Crotalus adamanteus*), PeerJ. (2017) 5, 10.7717/peerj.3249, 2-s2.0-85018364644. PMC5410154 28462047

[44] Hofmann E. P. , Rautsaw R. M. , Strickland J. L. et al., Comparative Venom-Gland Transcriptomics and Venom Proteomics of Four Sidewinder Rattlesnake (*Crotalus cerastes*) Lineages Reveal Little Differential Expression Despite Individual Variation, Scientific Reports. (2018) 8, no. 1, 10.1038/s41598-018-33943-5, 2-s2.0-85055079806. PMC6195556 30341342

[45] Margres M. J. , McGivern J. J. , Seavy M. , Wray K. P. , Facente J. , and Rokyta D. R. , Contrasting Modes and Tempos of Venom Expression Evolution in Two Snake Species, Genetics. (2015) 199, no. 1, 165–176, 10.1534/genetics.114.172437, 2-s2.0-84920885286. 25387465 PMC4286681

[46] Mason A. J. , Margres M. J. , Strickland J. L. , Rokyta D. R. , Sasa M. , and Parkinson C. L. , Trait Differentiation and Modular Toxin Expression in palm-pitvipers, BMC Genomics. (2020) 21, no. 1, 10.1186/s12864-020-6545-9. PMC7014597 32046632

[47] Mason A. J. , Holding M. L. , Rautsaw R. M. , Rokyta D. R. , Parkinson C. L. , and Gibbs H. L. , Yoder A. , Venom Gene Sequence Diversity and Expression Jointly Shape Diet Adaptation in Pitvipers, Molecular Biology and Evolution. (2022) 39, no. 4, 10.1093/molbev/msac082. PMC9040050 35413123

[48] Westfall A. K. , Gopalan S. S. , Perry B. W. et al., Yi S. , Single-Cell Heterogeneity in Snake Venom Expression Is Hardwired by Co-Option of Regulators From Progressively Activated Pathways, Genome Biology and Evolution. (2023) 15, no. 6, 10.1093/gbe/evad109. PMC10289209 37311204

[49] Han X. , Acerbi E. , Ye Q. , and Kini R. M. , Molecular Insights into the Regulation of Expression of Snake Venom Toxin Genes in *Pseudonaja textilis* , The FASEB Journal. (2016) 30, no. S1, 10.1096/fasebj.30.1_supplement.lb103.

[50] Gibbs H. L. , Sanz L. , and Calvete J. J. , Snake Population Venomics: Proteomics-Based Analyses of Individual Variation Reveals Significant Gene Regulation Effects on Venom Protein Expression in Sistrurus Rattlesnakes, Journal of Molecular Evolution. (2009) 68, no. 2, 113–125, 10.1007/s00239-008-9186-1, 2-s2.0-61349193761. 19184165

[51] Reyes-Velasco J. , Card D. C. , Andrew A. L. et al., Expression of Venom Gene Homologs in Diverse Python Tissues Suggests a New Model for the Evolution of Snake Venom, Molecular Biology and Evolution. (2015) 32, no. 1, 173–183, 10.1093/molbev/msu294, 2-s2.0-84922366053. 25338510

[52] Schield D. R. , Card D. C. , Hales N. R. et al., The Origins and Evolution of Chromosomes, Dosage Compensation, and Mechanisms Underlying Venom Regulation in Snakes, Genome Research. (2019) 29, no. 4, 590–601, 10.1101/gr.240952.118, 2-s2.0-85063988314. 30898880 PMC6442385

[53] Perry B. W. , Schield D. R. , Westfall A. K. , Mackessy S. P. , and Castoe T. A. , Physiological Demands and Signaling Associated With Snake Venom Production and Storage Illustrated by Transcriptional Analyses of Venom Glands, Scientific Reports. (2020) 10, no. 1, 10.1038/s41598-020-75048-y. PMC7582160 33093509

[54] Modahl C. M. , Han S. X. , Van Thiel J. et al., Distinct Regulatory Networks Control Toxin Gene Expression in Elapid and Viperid Snakes, BMC Genomics. (2024) 25, no. 1, 10.1186/s12864-024-10090-y. PMC10874052 38365592

[55] Durban J. , Sasa M. , and Calvete J. J. , Venom Gland Transcriptomics and microRNA Profiling of Juvenile and Adult Yellow-Bellied Sea Snake, Hydrophis Platurus, From Playa Del Coco (Guanacaste, Costa Rica), Toxicon. (2018) 153, 96–105, 10.1016/j.toxicon.2018.08.016, 2-s2.0-85052960967. 30189242

[56] Gopalan S. S. , Perry B. W. , Schield D. R. , Smith C. F. , Mackessy S. P. , and Castoe T. A. , Origins, Genomic Structure and Copy Number Variation of Snake Venom Myotoxins, Toxicon. (2022) 216, 92–106, 10.1016/j.toxicon.2022.06.014. 35820472

[57] Davies E. L. and Arbuckle K. , Coevolution of Snake Venom Toxic Activities and Diet: Evidence that Ecological Generalism Favours Toxicological Diversity, Toxins. (2019) 11, no. 12, 10.3390/toxins11120711. PMC6950196 31817769

[58] Schaeffer R. , Pascolutti V. J. , Jackson T. N. W. , and Arbuckle K. , Diversity Begets Diversity When Diet Drives Snake Venom Evolution, but Evenness Rather Than Richness is What Counts, Toxins. (2023) 15, no. 4, 10.3390/toxins15040251. PMC10142186 37104189

[59] Zheng H. , Wang J. , Fan H. et al., Arkhipova I. , Comparative Venom Multiomics Reveal the Molecular Mechanisms Driving Adaptation to Diverse Predator–Prey Ecosystems in Closely Related Sea Snakes, Molecular Biology and Evolution. (2023) 40, no. 6, 10.1093/molbev/msad125. PMC10265070 37279580

[60] Chandrasekara U. , Harris R. J. , and Fry B. G. , The Target Selects the Toxin: Specific Amino Acids in Snake-Prey Nicotinic Acetylcholine Receptors That are Selectively Bound by King Cobra Venoms, Toxins. (2022) 14, no. 8, 10.3390/toxins14080528. PMC9416539 36006190

[61] Li M. , Fry B. G. , and Kini R. M. , Putting the Brakes on Snake Venom Evolution: The Unique Molecular Evolutionary Patterns of *Aipysurus eydouxii* (Marbled Sea Snake) Phospholipase A2 Toxins, Molecular Biology and Evolution. (2005) 22, no. 4, 934–941, 10.1093/molbev/msi077, 2-s2.0-16344384549. 15635056

[62] Lynch V. J. , Inventing an Arsenal: Adaptive Evolution and Neofunctionalization of Snake Venom Phospholipase A2 Genes, BMC Evolutionary Biology. (2007) 7, no. 1, 10.1186/1471-2148-7-2, 2-s2.0-33846669778. PMC1783844 17233905

[63] Aird S. D. , Arora J. , Barua A. , Qiu L. , Terada K. , and Mikheyev A. S. , Population Genomic Analysis of a Pitviper Reveals Microevolutionary Forces Underlying Venom Chemistry, Genome Biology and Evolution. (2017) 9, no. 10, 2640–2649, 10.1093/gbe/evx199, 2-s2.0-85042163432. 29048530 PMC5737360

[64] Rao W. , Kalogeropoulos K. , Allentoft M. E. et al., The Rise of Genomics in Snake Venom Research: Recent Advances and Future Perspectives, GigaScience. (2022) 11, 10.1093/gigascience/giac024. PMC8975721 35365832

[65] Aird S. D. , Aggarwal S. , Villar-Briones A. , Tin M. M. Y. , Terada K. , and Mikheyev A. S. , Snake Venoms Are Integrated Systems, but Abundant Venom Proteins Evolve More Rapidly, BMC Genomics. (2015) 16, no. 1, 10.1186/s12864-015-1832-6, 2-s2.0-84940037418. PMC4552096 26315097

[66] Casewell N. R. , Wagstaff S. C. , Harrison R. A. , Renjifo C. , and Wuster W. , Domain Loss Facilitates Accelerated Evolution and Neofunctionalization of Duplicate Snake Venom Metalloproteinase Toxin Genes, Molecular Biology and Evolution. (2011) 28, no. 9, 2637–2649, 10.1093/molbev/msr091, 2-s2.0-79960307199. 21478373

[67] Sunagar K. , Johnson W. E. , O’Brien S. J. , Vasconcelos V. , and Antunes A. , Evolution of CRISPs Associated With Toxicoferan-Reptilian Venom and Mammalian Reproduction, Molecular Biology and Evolution. (2012) 29, no. 7, 1807–1822, 10.1093/molbev/mss058, 2-s2.0-84863583935. 22319140

[68] Giorgianni M. W. , Dowell N. L. , Griffin S. , Kassner V. A. , Selegue J. E. , and Carroll S. B. , The Origin and Diversification of a Novel Protein Family in Venomous Snakes, Proceedings of the National Academy of Sciences of the United States of America. (2020) 117, no. 20, 10911–10920, 10.1073/pnas.1920011117. 32366667 PMC7245079

[69] Mancuso M. , Zaman S. , Maddock S. T. et al., Resistance is Not Futile: Widespread Convergent Evolution of Resistance to Alpha-Neurotoxic Snake Venoms in Caecilians (Amphibia: Gymnophiona), Indian Journal of Management Science. (2023) 24, no. 14, 10.3390/ijms241411353. PMC10379402 37511112

[70] Jiang Y. , Li Y. , Lee W. et al., Venom Gland Transcriptomes of Two Elapid Snakes (*Bungarus multicinctus* and *Naja atra*) and Evolution of Toxin Genes, BMC Genomics. (2011) 12, no. 1, 10.1186/1471-2164-12-1, 2-s2.0-78650724989. PMC3023746 21194499

[71] Chong H. P. , Tan K. Y. , Tan N. H. , and Tan C. H. , Exploring the Diversity and Novelty of Toxin Genes in Naja Sumatrana, the Equatorial Spitting Cobra From Malaysia Through De Novo Venom-Gland Transcriptomics, Toxins. (2019) 11, no. 2, 10.3390/toxins11020104, 2-s2.0-85061505954. PMC6409529 30754700

[72] Xu N. , Zhao H. Y. , Yin Y. et al., Combined Venomics, Antivenomics and Venom Gland Transcriptome Analysis of the Monocoled Cobra (*Naja kaouthia*) from China, Journal of Proteomics. (2017) 159, 19–31, 10.1016/j.jprot.2017.02.018, 2-s2.0-85014791629. 28263888

[73] Manuwar A. , Dreyer B. , Böhmert A. et al., Proteomic Investigations of Two Pakistani Naja Snake Venoms Species Unravel the Venom Complexity, Posttranslational Modifications, and Presence of Extracellular Vesicles, Toxins. (2020) 12, no. 11, 10.3390/toxins12110669. PMC7690644 33105837

[74] Suryamohan K. , Krishnankutty S. P. , Guillory J. et al., The Indian Cobra Reference Genome and Transcriptome Enables Comprehensive Identification of Venom Toxins, Nature Genetics. (2020) 52, no. 1, 106–117, 10.1038/s41588-019-0559-8. 31907489 PMC8075977

[75] Junqueira-de-Azevedo I. L. M. , Bastos C. M. V. , Ho P. L. , Luna M. S. , Yamanouye N. , and Casewell N. R. , Venom-Related Transcripts From *Bothrops jararaca* Tissues Provide Novel Molecular Insights into the Production and Evolution of Snake Venom, Molecular Biology and Evolution. (2015) 32, no. 3, 754–766, 10.1093/molbev/msu337, 2-s2.0-84982163154. 25502939 PMC4327157

[76] Cardoso K. C. , Da Silva M. J. , Costa G. G. et al., A Transcriptomic Analysis of Gene Expression in the Venom Gland of the Snake Bothrops alternatus (Urutu), BMC Genomics. (2010) 11, no. 1, 10.1186/1471-2164-11-605, 2-s2.0-77958132097. PMC3017861 20977763

[77] Freitas-de-Sousa L. A. , Nachtigall P. G. , Portes-Junior J. A. et al., Size Matters: An Evaluation of the Molecular Basis of Ontogenetic Modifications in the Composition of *Bothrops jararacussu* Snake Venom, Toxins. (2020) 12, no. 12, 10.3390/toxins12120791. PMC7763748 33322460

[78] Augusto-de-Oliveira C. , Stuginski D. R. , Kitano E. S. et al., Dynamic Rearrangement in Snake Venom Gland Proteome: Insights into *Bothrops jararaca* Intraspecific Venom Variation, Journal of Proteome Research. (2016) 15, no. 10, 3752–3762, 10.1021/acs.jproteome.6b00561, 2-s2.0-84990837295. 27575776

[79] Tse T. C. , Tsai I. H. , Chan Y. Y. , and Tsai T. S. , Venom Proteomics of *Trimeresurus gracilis*, a Taiwan-Endemic Pitviper, and Comparison of Its Venom Proteome and VEGF and CRISP Sequences with Those of the Most Related Species, Toxins. (2023) 15, no. 7, 10.3390/toxins15070408. PMC10467061 37505677

[80] Nakashima K. , Ogawa T. , Oda N. et al., Accelerated Evolution of *Trimeresurus flavoviridis* Venom Gland Phospholipase A2 Isozymes, Proceedings of the National Academy of Sciences of the United States of America. (1993) 90, no. 13, 5964–5968, 10.1073/pnas.90.13.5964, 2-s2.0-0027253951. 8327468 PMC46847

[81] Chijiwa T. , Deshimaru M. , Nobuhisa I. et al., Regional Evolution of Venom-Gland Phospholipase A2 Isoenzymes of *Trimeresurus flavoviridis* Snakes in the Southwestern Islands of Japan, Biochemical Journal. (2000) 347, no. 2, 491–499, 10.1042/bj3470491. 10749679 PMC1220982

[82] Nakashima K. , Nobuhisa I. , Deshimaru M. et al., Accelerated Evolution in the Protein-Coding Regions is Universal in Crotalinae Snake Venom Gland Phospholipase A2 Isozyme Genes, Proceedings of the National Academy of Sciences of the United States of America. (1995) 92, no. 12, 5605–5609, 10.1073/pnas.92.12.5605, 2-s2.0-0029014297. 7777556 PMC41745

[83] Tsai I. H. , Wang Y. M. , Chen Y. H. , Tsai T. S. , and Tu M. C. , Venom Phospholipases A2 of Bamboo Viper (*Trimeresurus stejnegeri*): Molecular Characterization, Geographic Variations and Evidence of Multiple Ancestries, Biochemical Journal. (2004) 377, no. 1, 215–223, 10.1042/bj20030818, 2-s2.0-1642494592. 12959640 PMC1223832

[84] Post Y. , Puschhof J. , Beumer J. et al., Snake Venom Gland Organoids, Cell. (2020) 180, no. 2, 233–247.e21, 10.1016/j.cell.2019.11.038. 31978343

[85] Puschhof J. , Post Y. , Beumer J. et al., Derivation of Snake Venom Gland Organoids for In Vitro Venom Production, Nature Protocols. (2021) 16, no. 3, 1494–1510, 10.1038/s41596-020-00463-4. 33504990

[86] Kaas Q. and Craik D. , Bioinformatics-Aided Venomics, Toxins. (2015) 7, no. 6, 2159–2187, 10.3390/toxins7062159, 2-s2.0-84934970617. 26110505 PMC4488696

[87] von Reumont B. M. , Anderluh G. , Antunes A. et al., Modern Venomics—Current Insights, Novel Methods, and Future Perspectives in Biological and Applied Animal Venom Research, GigaScience. (2022) 11, 10.1093/gigascience/giac048. PMC9155608 35640874

[88] Kusuma W. A. , Fadli A. , Fatriani R. et al., Prediction of the Interaction Between *Calloselasma rhodostoma* Venom-Derived Peptides and Cancer-Associated Hub Proteins: A Computational Study, Heliyon. (2023) 9, no. 11, 10.1016/j.heliyon.2023.e21149. PMC10637925 37954374

[89] Mouchbahani-Constance S. and Sharif-Naeini R. , Proteomic and Transcriptomic Techniques to Decipher the Molecular Evolution of Venoms, Toxins. (2021) 13, no. 2, 10.3390/toxins13020154. PMC7920473 33669432

[90] Ojeda P. , Ramírez D. , Alzate-Morales J. , Caballero J. , Kaas Q. , and González W. , Computational Studies of Snake Venom Toxins, Toxins. (2017) 10, no. 1, 10.3390/toxins10010008, 2-s2.0-85038907913. PMC5793095 29271884

[91] Maghfira A. , Auliya I. , Nuringtyas T. R. et al., Exploring Venom-Derived Peptides From *Calloselasma rhodostoma* Snake as Promising Cholinesterase Inhibitors for Alzheimer’s Disease Therapy, Pertanika Journal of Tropical Agricultural Science. (2025) 48, no. 6, 10.47836/jtas.48.6.21.

[92] Kusuma W. A. , Fadli A. , Amanda C. N. et al., In Silico Targeting of Snake Venom Phospholipase A2 Using Phytochemicals From *Cyanthillium cinereum*: An Integrated Machine Learning and Molecular Simulation Study, Journal of Applied Pharmaceutical Science. (2026) 10.7324/JAPS.2026.271166.

[93] Schendel V. , Rash L. D. , Jenner R. A. , and Undheim E. A. , The Diversity of Venom: The Importance of Behavior and Venom System Morphology in Understanding Its Ecology and Evolution, Toxins. (2019) 11, no. 11, 10.3390/toxins11110666. PMC6891279 31739590

[94] Siqueira‐Silva T. , De Lima L. A. G. , Chaves‐Silveira J. et al., Ecological and Biogeographic Processes Drive the Proteome Evolution of Snake Venom, Global Ecology and Biogeography. (2021) 30, no. 10, 1978–1989, 10.1111/geb.13359.

[95] Sunagar K. , Morgenstern D. , Reitzel A. M. , and Moran Y. , Ecological Venomics: How Genomics, Transcriptomics and Proteomics Can Shed New Light on the Ecology and Evolution of Venom, Journal of Proteomics. (2016) 135, 62–72, 10.1016/j.jprot.2015.09.015, 2-s2.0-84960813416. 26385003

